# Role of the *kdpDE* Regulatory Operon of *Mycobacterium tuberculosis* in Modulating Bacterial Growth *in vitro*

**DOI:** 10.3389/fgene.2021.698875

**Published:** 2021-07-29

**Authors:** Moloko C. Cholo, Maborwa T. Matjokotja, Ayman G. Osman, Ronald Anderson

**Affiliations:** Department of Immunology, Faculty of Health Sciences, University of Pretoria, Pretoria, South Africa

**Keywords:** *Mycobacterium tuberculosis*, two-component system KdpDE, KdpFABC system, Trk system, K^+^-uptake systems, K^+^ concentration, pH level, gene expression

## Abstract

Bacteria use K^+^-uptake transporters differentially for adaptation in varying growth conditions. In *Mycobacterium tuberculosis*, two K^+^-uptake systems, the Trk comprising the CeoB and CeoC proteins and the Kdp consisting of the two-component system (TCS), KdpDE and KdpFABC, have been characterized, but their selective utilization during bacterial growth has not been completely explored. In the current study, the roles of the *M. tuberculosis* KdpDE regulatory system alone and in association with the Trk transporters in bacterial growth were investigated by evaluating the growth of *M. tuberculosis* KdpDE-deletion and KdpDE/Trk (KT)-double knockout mutant strains in planktonic culture under standard growth conditions. The KT-double knockout mutant strain was first constructed using homologous recombination procedures and was evaluated together with the KdpDE-deletion mutant and the wild-type (WT) strains with respect to their rates of growth, K^+^-uptake efficiencies, and K^+^-transporter gene expression during planktonic growth. During growth at optimal K^+^ concentrations and pH levels, selective deletion of the TCS KdpDE (KdpDE-deletion mutant) led to attenuation of bacterial growth and an increase in bacterial K^+^-uptake efficiency, as well as dysregulated expression of the *kdpFABC* and *trk* genes. Deletion of both the KdpDE and the Trk systems (KT-double knockout) also led to severely attenuated bacterial growth, as well as an increase in bacterial K^+^-uptake efficiency. These results demonstrate that the KdpDE regulatory system plays a key role during bacterial growth by regulating K^+^ uptake *via* modulation of the expression and activities of both the KdpFABC and Trk systems and is important for bacterial growth possibly by preventing cytoplasmic K^+^ overload.

## Introduction

Growth of *Mycobacterium tuberculosis* is optimal in artificial culture media supplemented with potassium (K^+^) concentrations of around 14–15 millimolar (mM) at a pH of about 6.8 (Piddington et al., 2000; Cholo et al., 2015; Baker et al., 2019; Salina et al., 2019). *Mycobacterium tuberculosis* uses K^+^ to support cellular metabolic activities, such as cell wall biosynthesis, protein synthesis, lipid metabolism, and aerobic respiration, which are associated with logarithmic growth (Salina et al., 2014). However, in K^+^-limiting (Salina et al., 2014) and low pH environments (pH 5.5; Cholo et al., 2015), this bacterial pathogen alters its metabolic rates, transitioning to slow-growing, persistent-to-non-growing, and dormant phenotypes and allowing for bacterial survival in these unfavorable growth conditions.

Other studies have shown that bacteria adapt to these adverse growth conditions through differential utilization of K^+^-uptake transport systems (Epstein, 2003). For example, *Escherichia coli* utilizes the Trk system at elevated K^+^ concentrations in near-neutral pH, while the K^+^-uptake permease (Kup: TrkD) is used at low pH. However, at K^+^-limiting conditions at neutral pH, *E. coli* utilizes the Kdp system (Roe et al., 2000; Epstein, 2003). In this context, *M. tuberculosis* possesses two active K^+^-uptake transporters, namely the Trk and Kdp systems (Cole et al., 1998). The Trk consists of two TrkA proteins, CeoB and CeoC, encoded by highly homologous *trk* genes (*ceoB* and *ceoC*) of the *ceoBC* operon. We have previously shown that the Trk system is constitutively expressed in *M. tuberculosis* (Cholo et al., 2015), and has a lower affinity for K^+^ than the Kdp (Cholo et al., 2006). It plays a role in slowing bacterial growth in optimal conditions in standard 7H9 broth medium (15 mM K^+^, pH 6.8; Cholo et al., 2006). The Trk system has also been implicated in bacterial dormancy, with the CeoB protein being expressed at low K^+^ concentrations in biofilm cultures (Kerns et al., 2014; Hegde, 2020), while both *trk* genes are upregulated at low extracellular pH levels in planktonic culture (Cholo et al., 2015).

The *M. tuberculosis* Kdp system, on the other hand, is an inducible two-component system (TCS) comprised of the KdpDE sensor-regulator and the high-affinity K^+^-uptake transporter complex, KdpFABC (Cholo et al., 2006). The two Kdp components consist of six proteins encoded by a cluster of six genes, arranged in two operons, *kdpDE* and *kdpFABC*. These operons are divergently transcribed on the bacterial genome; being separated by an intergenic region (~234 bp) located between the *kdpD* and *kdpF* genes (Cole et al., 1998; Cholo et al., 2008). Activation of the Kdp system is mediated by the KdpDE, consisting of the sensor kinase KdpD and response regulator KdpE proteins (Steyn et al., 2003; Freeman et al., 2013). The KdpD and KdpE proteins interact with one another under basal conditions, with KdpD sensing the environmental stimulus and undergoing autophosphorylation at the histidine-642 residue, transferring the phosphoryl moiety to the KdpE subunit at the aspartate-52 residue, resulting in its phosphorylation, and leading to induction of the *kdpFABC* operon (Steyn et al., 2003; Agrawal and Saini, 2014). Low extracellular pH levels (Cholo et al., 2015), as well as K^+^-limiting conditions (Salina et al., 2014), are environmental stressors that lead to the induction of both the *kdpDE* and *kdpFABC* operons. These adverse growth conditions also result in acquisition of a dormant phenotype, seemingly implicating the involvement of the Kdp system in bacterial survival.

As with growth in adverse conditions of low extracellular K^+^ and pH, information on the role of the Kdp system during mycobacterial growth in optimal K^+^ concentrations and pH levels *in vitro* is also limited. In this context, we have previously reported that in the setting of optimal growth conditions, the Kdp system is repressed when the Trk system is functional, being induced and activated as a back-up when the Trk system is inactive (Cholo et al., 2006). Despite the limited information on growth, a few studies have identified that only the *kdpE* among the *kdp* genes, is necessary for optimal growth of *M. tuberculosis* (Sassetti et al., 2003; Griffin et al., 2011), as well as that of *Mycobacterium smegmatis* (Ali et al., 2017).

The issue of the differential utilization of these high- and low-affinity K^+^-uptake systems of *M. tuberculosis* during planktonic growth of the pathogen, including the seemingly modulatory role of KdpDE, have been explored in the current study. The research strategy used was based on a comparison of the growth of a selective KdpDE-deletion mutant strain and a recently constructed KdpDE/Trk (CeoBC; KT)-double knockout mutant strain with that of the wild-type (WT) strain of *M. tuberculosis* in standard growth conditions.

## Materials and Methods

### Antimicrobial Agents and Chemicals

Unless indicated, all chemicals were purchased from the Sigma Chemical Co (St. Louis, MO, United States). Kanamycin and hygromycin antibiotics were used at 10 and 50 mg/L, respectively, for the selection of antibiotic-resistant colonies; 5-bromo-4-chloro-3-indolyl-B-D-galactoside (X-Gal) at 0.24 mg/L for blue colonies and sucrose at 2% (20 g/L) as a counter-selectable marker for *sacB*-expressing clones.

Rubidium-86 chloride (^86^Rb^+^) was purchased from PerkinElmer Life and Analytical Sciences, Du Pont-NEN Research Products, Boston, MA, United States.

### Strains and Growth Media

All plasmids and bacterial strains used in this study are as shown in Table 1. The *E. coli* DH5α competent cells were used for cloning procedures for plasmid transformation. The pSOUP42 suicide-delivery vector (SDV) carrying the mutated *M. tuberculosis kdpDE* fragment and the KdpDE-deletion mutant strain of *M. tuberculosis*, were kindly provided by Professor N. Stoker, Royal Veterinary College, United Kingdom (Parish et al., 2003). The *M. tuberculosis* Trk-deletion mutant strain, which we had constructed previously (Cholo et al., 2006) was used for the construction of the KT-double knockout. The *M. tuberculosis* KdpDE-deletion (Parish et al., 2003) and the KT-double knockout (current study) mutant strains were compared with the WT strain for phenotypic and genotypic characteristics.

**Table 1:** Plasmids and bacterial strains used in the study.

Strain	Feature or relevant genotype	Source
Plasmids		
pSOUP42	*Pac*I fragment from pGOAL19, unmarked 1,691-bp *Sph*I *kdpDE* deletion in *kdpD* and *kdpE* genes.	Parish et al., 2003
pSOUP43	*Pac*I fragment from pGOAL17, unmarked 1,691-bp *Sph*I *kdpDE* deletion in *kdpD* and *kdpE* genes.	This study
Bacteria		
DH5α	Wild type *Escherichia coli* strain.	
H37Rv	Wild type laboratory *M. tuberculosis* strain ATCC 25618.	Parish et al., 2003
KdpDE-deletion	Deletion of 1,691-bp *Sph*I *kdpDE* fragment in *kdpD* and *kdpE* genes.	Parish et al., 2003
Trk-deletion	Marked mutant, deletion of 348-bp *ceoB*, insertion of 1746-bp *hyg*-resistant gene cassette at *ceoC* gene.	Cholo et al., 2006
KdpDE/Trk-double knockout mutant	Marked mutant, combined mutations of *kdpDE* and *ceoBC* operons.	This study

The Psi (Ψ)- broth medium (5 g Bacto yeast extract, 20 g Bacto tryptone, 5 g MgSO_4_/L, and pH 7.5) was used for preparation of competent *E. coli* cells and Luria-Bertani (LB) broth for growing plasmid-carrying *E. coli* bacteria, while the *E. coli* colonies were developed on Luria agar (LA) medium. For *M. tuberculosis* cultures, 7H9 broth and 7H10 agar (Difco) media supplemented with 10% oleic acid, dextrose, catalase (OADC), and 2/5% glycerol with/without 0.05% Tween 80 respectively, were used for liquid-based growth assays and colony development, respectively.

### Construction of a KdpDE/Trk (CeoBC)-Double Knockout Mutant

The KT-deletion mutant, which is characterized by inactivation of both the *kdpDE* and *trk* (*ceoBC*) operons, was constructed using homologous recombination following a two-step strategy (Parish and Stoker, 2000). Prior to electroporation, the *hyg*, P_Ag85_-*lacZ*, P_hsp60_-*sacB PacI* cassette in the pSOUP42 SDV was replaced with the P_Ag85_-*lacZ*, P_hsp60_-*sacB PacI* cassette from pGOAL17 to form pSOUP43 (Table 1). Briefly, approximately 5 μg of UV-pretreated pSOUP43 was electroporated into the *M. tuberculosis* Trk-deletion mutant, carrying the mutation of the *ceoBC* operon (Cholo et al., 2006) and plated onto 7H10 agar medium supplemented with hygromycin, kanamycin, and X-Gal for the isolation of blue single crossover (SCO) clones, followed by isolation of white double-crossovers (DCOs) on non-selection media. The DCO clones were characterized for absence of the plasmid phenotypically using sucrose and kanamycin sensitivity testing procedures. DNA samples from white sucrose-resistant, kanamycin-sensitive *M. tuberculosis* clones were extracted and mutations at the *kdpDE* and *ceoBC* operons were confirmed by a non-radioactive Southern blotting procedure using a digoxigenin (DIG)-labeled PCR-synthesized probe as described in the PCR DIG Probe Synthesis kit (Roche Molecular Biochemicals, Mannheim, Germany).

### Bacterial Inoculum Preparation

A bacterial inoculum of each strain was prepared as described, with minor modifications (Cholo et al., 2015; Mothiba et al., 2015). Briefly, a seed culture of *M. tuberculosis* cells was inoculated into 50 ml of 7H9 broth and grown to the mid-log phase at 37°C under stirring conditions. The bacterial cells were harvested by centrifugation at 2851 × *g* at room temperature (RT) for 15 min and the supernatant discarded. The pellet was washed twice and re-suspended in 7H9 broth, followed by adjustment of the optical density (OD) to 1.2 at 540 nm, yielding ca. 10^8^–10^9^ colony-forming units (cfu)/ml. An inoculum of ca. 10^5^ cfu/ml was used in all of the assays.

### Preparation of Bacterial Cultures at Logarithmic Phases

For each strain, the bacterial cultures were prepared by inoculating approximately 10^5^ cfu/ml cells into 7H9 broth followed by incubation of the culture at 37°C under stirring conditions until the early-, mid-, and late-log phases were reached, corresponding to ODs of 0.1–0.3, 0.4–0.6, and 2.0–2.3 at 540 nm, respectively (Cholo et al., 2015).

### Rates of Growth

Cultures for determination of the rates of growth were prepared by inoculating ca. 10^5^ cfu/ml of cells of the WT and mutant strains of *M. tuberculosis* into 7H9 broth. The cultures were thoroughly mixed followed by incubation at 37°C for 15 days in the dark with continuous stirring. The cultures were sampled every 3 days beginning at day 0 (D0) to day 15 (D15) and growth was determined spectrophotometrically at a wavelength of 540 nm. The rates of growth of each strain were determined as the time taken by the bacteria to reach the different logarithmic growth phases.

### Uptake of Rubidium (^86^Rb^+^)

Uptake of K^+^ by the WT and mutant strains was determined at the mid- and late-log phases using ^86^Rb^+^ as a surrogate tracer for K^+^. Briefly, the bacteria were harvested from cultures grown to the two logarithmic growth phases and resuspended to ca. 10^6^ cfu/ml in K^+^-free buffer (KONO) containing 2 mCi/L ^86^Rb^+^ and uptake of the radioisotope determined as absolute counts per minute (cpm) as previously described (Steel et al., 1999; Cholo et al., 2015).

### Extracellular Potassium and pH

The K^+^ concentrations and pH levels were determined at the early-, mid-, and late-log phases for each strain. Following culture preparation, the supernatants were harvested by centrifugation (2,851 × *g*, 15 min) followed by decontamination by heat treatment at 95°C for 60 min. The K^+^ concentrations and pH levels were measured in the undiluted samples by indirect potentiometry utilizing a K^+^-selective electrode in conjunction with a Na^+^-reference electrode using the Beckman Coulter Synchron LX 20 System (Beckman Coulter, Ireland Inc., Gateway, Ireland) and the Crison micropH2001 pH meter (Crison Instruments, Barcelona, Spain), respectively. These measurements were determined at the initial (D0), intermediate and final (early-, mid-, and late-log) growth phases, as well as in the processed and unprocessed bacteria-free 7H9 broth medium.

### Gene Expression Using the Reverse-Transcriptase-PCR

Gene expression was performed at the early-, mid-, and late-log growth phases in standard 7H9 liquid culture medium as described previously (Cholo et al., 2015). Briefly, RNA was extracted following the Trizol method, and complementary deoxyribonucleic acid (cDNA) was synthesized using the Sigma Enhanced Avian HS reverse transcriptase-PCR (RT-PCR) kit and amplified by quantitative (q)PCR using the LightCycler FastStart DNA Master SYBR Green I kit with the LightCycler 2.0 instrument (Roche Molecular Biochemicals, Mannheim, Germany). The quantities of the individual genes were determined using absolute (AQ) and relative (RQ) quantifications with *sigA* as the reference gene. The relative quantifications were determined based on quantification cycles (Cq) using the 2^−ΔΔCq^ method.

### Statistical Analysis

All statistical analyses were performed using the INSTAT program and the unpaired and paired Student *t*-test/Mann–Whitney U-test for analysis of growth rates and gene expression data, respectively. The results are expressed as the means ± SDs. Significance levels were taken at a *p* ≤ 0.05.

## Results

### Construction of the KT-Double Knockout Mutant

In order to investigate the roles of the TCS KdpDE system on bacterial growth, alone and in association with the Trk system, acquisition of the K^+^-uptake mutant strains lacking the single KdpDE regulatory system (KdpDE-deletion mutant strain), as well as a combination of both the KdpDE and Trk systems (KT-double knockout mutant strain), was necessary.

The TCS KdpDE, together with the KdpFABC transporter, are the main components of the Kdp system, encoded by separate *kdpDE* and *kdpFABC* operons, transcribed in opposite directions, with the start codon of *kdpD* separated by 234 bp from the start codon of *kdpF*. This genomic arrangement of the *kdp* operons is similar to that of *Mycobacterium bovis*, but is different from those of other bacterial and mycobacterial species, which show both operons having similar transcriptional orientations with the *kdpD* being adjacent to the *kdpC* (Cole et al., 1998; Cholo et al., 2008; Agrawal and Saini, 2014).

The construction of the single KdpDE-deletion mutant of *M. tuberculosis* was as previously reported (Parish et al., 2003). Both the *kdpD* (*Rv1028c*: 2583 bp) and *kdpE* (*Rv1027c*: 681 bp) genes are transcribed in the negative direction with *kdpE* located at position 1148.427–1149.107 (681 bp) with its start codon overlapping the stop codon of *kdpD* found at position 1149.104–1051.686 on the chromosome. Mutation of the *kdpDE* operon was achieved by deletion of a 1,691-bp *Sph*I fragment that spans both the *kdpD* and *kdpE* genes, resulting in inactivation of the sensor kinase/response regulator of the KdpFABC system. However, due to separation of the *kdpDE* and promoter-carrying *kdpFABC* operons on the *M. tuberculosis* genome, the promoter region of the *kdpFABC* operon remained genotypically intact.

**Figure 1:**
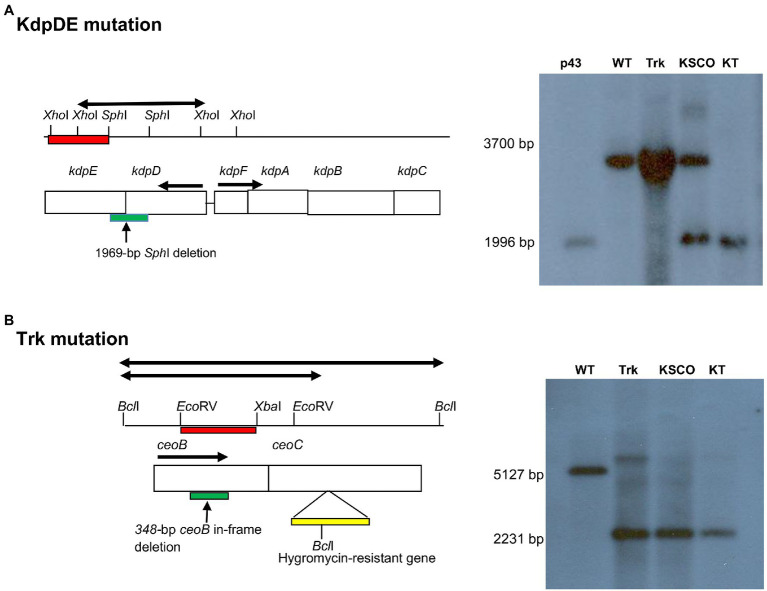
Schematic illustrations of allelic exchange mutagenesis of the *kdpDE* and *ceoBC* mutations in the KT-double knockout strain of *M. tuberculosis* as shown previously for inactivation of the single Trk- and KdpDE-deletion mutant strains (Parish et al., 2003; Cholo et al., 2006). The maps were not drawn to scale. DNA samples were digested with **(A)** *Xho*I and probed with 1,010-bp *kdpDE* PCR-synthesized fragments (red bar) for detection of the 1996-bp *Xho*I-*kdpDE* mutated fragment (thick black arrow) and **(B)** *Bcl*I and probed with 714-bp *ceoB* PCR-synthesized fragment (red bar) for detection of the 2,231-bp *Bcl*I-*ceoBC* mutated fragment (thick black arrow). Deletions in *kdpDE* and *ceoB* genes are shown by green bars, while insertion of the *hyg* gene in *ceoC* gene is shown by yellow bar. p43, pSOUP43; WT, wild type; Trk, Trk-deletion; KSCO, *kdpDE* single crossover; KT, KT-double knockout.

In the case of the KT-double knockout strain of *M. tuberculosis*, we used the Trk-deletion mutant strain, which we had constructed previously (Cholo et al., 2006). The *trk* genes comprising the *ceoB* (*Rv2691*: 684 bp) and *ceoC* (*Rv2692*: 663 bp) genes are found on the *ceoBC* operon, with the *ceoB* located at position 3009.344–3010.027, with its stop codon overlapping the start codon of *ceoC* found at position 3010.024–3010.686 on the chromosome. Both *trk* genes are transcribed in the positive direction. The Trk-deletion mutant strain was characterized by inactivation of both the *trk* genes, resulting in a 348-bp deletion at *ceoB* gene and insertion of the 1746-bp *Bam*HI-*Bgl*II *hyg* resistance cassette, derived from the plJ963 vector, at the *Nhe*I site of the *ceoC* gene. The KT-double knockout mutant was constructed by introducing a *kdpDE*-deletion fragment-carrying plasmid, pSOUP43, constructed as described (Parish et al., 2003; Table 1) into the Trk-deletion mutant (Cholo et al., 2006; Figure 1). Successful mutagenesis of the *kdpDE* operon in the KT-double knockout mutant strain was evident by detection of the 1996-bp *Xho*I-*kdpDE* fragment with the 1,010-bp PCR-synthesized *kdpDE* probe (Figure 1A; Table 2; Parish et al., 2003). For the *ceoBC* operon, mutation was revealed by detection of the 2,231-bp *Bcl*I-*ceoBC* fragment using the 714-bp PCR-synthesized *ceoB* probe (Figure 1B; Table 2; Cholo et al., 2006).

**Table 2:** Primers used for probe preparation for Southern blotting for KT-double knockout mutant strain construction.

Gene name	Forward primer (bp)	Reverse primer (bp)	Target fragment length (bp)
*kdpDE*	TCG AGC CCG CAC TGC GCA CCG TGC CGC TGG (30)	CTG GAA ATG CTG GCC CGC AAC CGC GGC AAG (30)	1,010
*ceoBC*	CCA TCA GGG CGC TGG CAA (18)	CGG CCT GTA GGA CCG TCT (18)	714

### Rates of Growth

**Figure 2:**
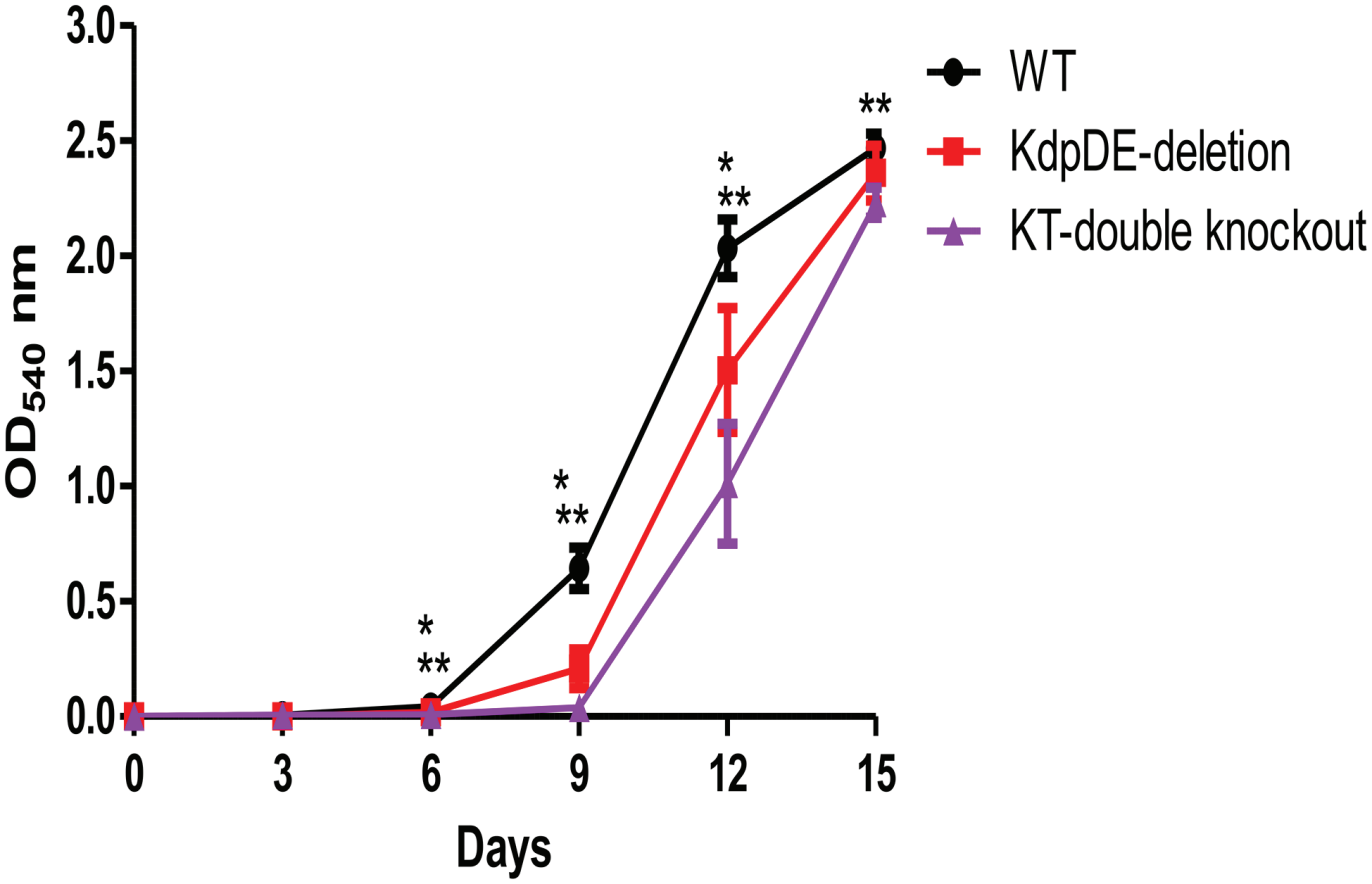
The rates of planktonic growth of the WT and mutant strains of *M. tuberculosis* measured over 15 days. The results are of three different experiments and are expressed as the mean optical density (OD) ± SD at 540 nm. The numbers of bacteria were 8.8 × 10^4^ ± 7.5 × 10^4^, 2.1 × 10^5^ ± 1.8 × 10^5^, and 9.7 × 10^4^ ± 1.3 × 10^5^ cfu/ml for the WT, KdpDE-deletion and KT-double knockout mutant strains at D0, respectively. The OD values for the WT, KdpDE-deletion and KT-double knockout mutants at D6 were: 0.044 ± 0.024, 0.018 ± 0.006, and 0.009 ± 0.006; at D9: 0.644 ± 0.09, 0.208 ± 0.084, and 0.04 ± 0.006; at D12: 2.034 ± 0.126, 1.505 ± 0.269, and 1.011 ± 0.259; and at D15: 2.468 ± 0.0064, 2.360 ± 0.118, and 2.232 ± 0.065, respectively. * and ** represent *p* ≤ 0.05 for the KdpDE-deletion and KT-double knockout mutants in relation to the WT, respectively. *Values of *p* between the WT and KdpDE-deletion mutant at D6, D9, and D12 were 0.026, 0.0022, and 0.0022 respectively; **Values of *p* between the WT and KT-double knockout at D6, D9, D12, and D15 were 0.0022 at each time point; Values of *p* between the KdpDE-deletion and KT-double knockout at D6, D9 and D12 were 0.0246, 0.0022, and 0.0260, respectively.

We used the WT and mutant strains to determine the role of the TCS KdpDE alone and in association with the Trk system on bacterial growth. This was achieved by assessing the rates of growth of the WT and the K^+^-uptake mutant strains in 7H9 broth medium (15 mM K^+^, pH 6.7) under aerobic conditions, sampled every 3 days beginning at D0 to D15 for OD determination. We have previously shown that the OD measurements of 0.1–0.3, 0.4–0.6, and 2.0–2.3 at 540 nm corresponded to the early-, mid-, and late-log phases, respectively (Cholo et al., 2015). The rate of growth of each strain was determined as the time, measured in days, taken by the bacteria to reach these growth phases, and the results are shown in Figure 2.

The numbers of bacteria were determined at D0 and were shown to be 8.8 × 10^4^ ± 7.5 × 10^4^, 2.1 × 10^5^ ± 1.8 × 10^5^, and 9.7 × 10^4^ ± 1.3 × 10^5^ cfu/ml for the WT, KdpDE-deletion, and KT-double knockout mutant strains, respectively. The results showed that the rates of growth of the WT and the mutant strains were different, entering the three log phases of growth at varying time points. The growth rates of the mutant strains were attenuated, showing prolonged early-log phases and reaching the early-, mid-, and late-log phases at D9, D12, and D15, respectively, while the WT reached these phases of growth at D6, D9, and D12. The rates of growth were significantly different between the WT and KdpDE-deletion mutant strain at D6, D9, and D12 (*p* < 0.05), while the rates of growth were comparable between the two strains at D15 (ODs: 2.468 ± 0.0064 and 2.360 ± 0.118 for the WT and KdpDE-deletion mutant, respectively, *p* = 0.132). However, the KT-double knockout strain was highly attenuated for growth, with the rates of growth of this mutant being significantly slower than those of the WT at D6, D9, D12, and D15 (*p* < 0.05).

Despite the KdpDE-deletion and KT-double knockout mutant strains reaching the early-, mid-, and late-log phases at the same time points (i.e., at D9, D12, and D15, respectively), the growth levels of the two mutant strains determined by comparing their OD measurements at the different time points, were nevertheless different, as shown in Figure 2, with the rate of growth of the KT-double knockout mutant strain at D6, D9, and D12 being significantly slower than that of the KdpDE-deletion mutant strain (*p* < 0.05).

As we have previously shown that *M. tuberculosis* utilizes the Trk K^+^-uptake transporter exclusively when cultured during standard growth conditions in 7H9 medium, suppressing the activity of the KdpFABC transporter (Cholo et al., 2006, 2015), these observations appear to implicate the KdpDE system in harmonizing the activities of both the Trk and KdpFABC systems to achieve optimum growth.

### ^86^Rb^+^ Uptake

**Figure 3:**
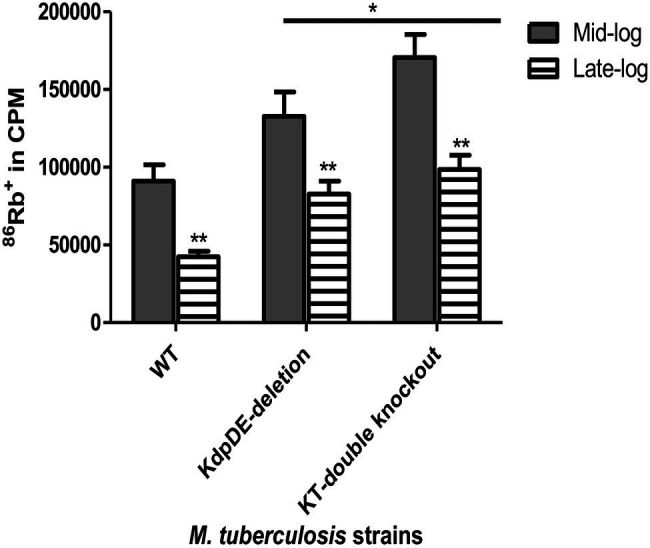
Rubidium (^86^Rb^+^) uptake by the WT and the K^+^-uptake-deletion mutant strains at the mid- and late-log phases. The results are of five experiments with three replicates for each time point represented as absolute counts per minute (cpm). The gray and striped bars represent uptake of ^86^Rb^+^ at the mid- and late-log phases, respectively. The cpm values were 91,041 ± 10464.87, 132748.3 ± 15,650, and 170577.3 ± 4877.88 at the mid-log phase and 42431.48 ± 3447.031, 82737.71 ± 8308.834, and 98569.04 ± 9211.451 at the late-log phase for H37Rv (WT), KdpDE-deletion, and KT-double knockout strains, respectively. Statistical differences at *p* < 0.05 are represented by * and **. *Values of *p* represent comparison of the responses of the WT strain relative to those of the mutant strains at the mid- and late-log phases and were 0.0397 and 0.0006 for the KdpDE-deletion and KT-double knockout mutant strains, respectively at the mid-log phase, while they were 0.0001 for both mutant strains at the late-log phase. The **values of *p* represent comparison of responses in each strain between mid- and late-log phases.

Following determination of growth rates, we then assessed the magnitudes of K^+^-uptake by measuring the uptake of ^86^Rb^+^ by the WT and mutant strains at the mid- and late-log phases of growth as described previously (Steel et al., 1999; Cholo et al., 2015) and the results are shown in Figure 3.

Using the ^86^Rb^+^-uptake model we have, previously shown that the Trk system has a low-affinity for K^+^ being responsible for K^+^ influx during bacterial growth at the mid- and late-log phases. On the other hand, the Kdp system has been characterized as the high-affinity K^+^ transporter, which is suppressed during the logarithmic phases of growth, being induced as a backup when the Trk system is not active (Cholo et al., 2006, 2015).

Somewhat surprisingly, the results revealed that the ^86^Rb^+^-uptake efficiencies of the mutant strains, were higher than those of the WT strain at both the mid- and late-log phases being highest at the mid-log phase for the KT-double knockout mutant (Figure 3). At the late-log phase, the ^86^Rb^+^-uptake efficiencies of the mutant strains were again higher than that of the WT strain, but not significantly different from one another (82,738 ± 8,309 and 98,569 ± 9,212 cpm for the KdpDE-deletion and KT-double knockout mutant strains, respectively, *p* = 0.126). These findings indicate that deletion of the KdpDE system results in K^+^ overload, presumably due to dysregulation of the Trk and KdpFABC K^+^ transporters, an event that may disrupt mycobacterial cytoplasmic pH, cellular metabolism, and growth.

For all the strains, the ^86^Rb^+^-uptake efficiencies were significantly reduced at the late-log phase, being 46, 62, and 57% of the corresponding efficiencies noted at the mid-log phase for the WT, KdpDE-deletion and KT-double knockout mutant strains, respectively, most probably due to decreased extracellular pH (Cholo et al., 2015).

### Extracellular Potassium Concentrations and pH Levels

Alterations in the extracellular K^+^ concentrations and pH levels that occur during the early-, mid-, and late-log phases of growth of the WT and mutant strains of *M. tuberculosis* under optimal culture conditions in 7H9 broth (15 mM K^+^ and pH 6.8) were determined. In the case of the extracellular K^+^ concentrations of the culture supernatants, these remained unchanged (14–15.5 mM) with respect to all three strains of *M. tuberculosis* and were comparable during the three log phases (Supplementary Table S1). The lack of significant alterations in extracellular K^+^ may reflect recycling of K^+^ during bacterial growth.

**Figure 4:**
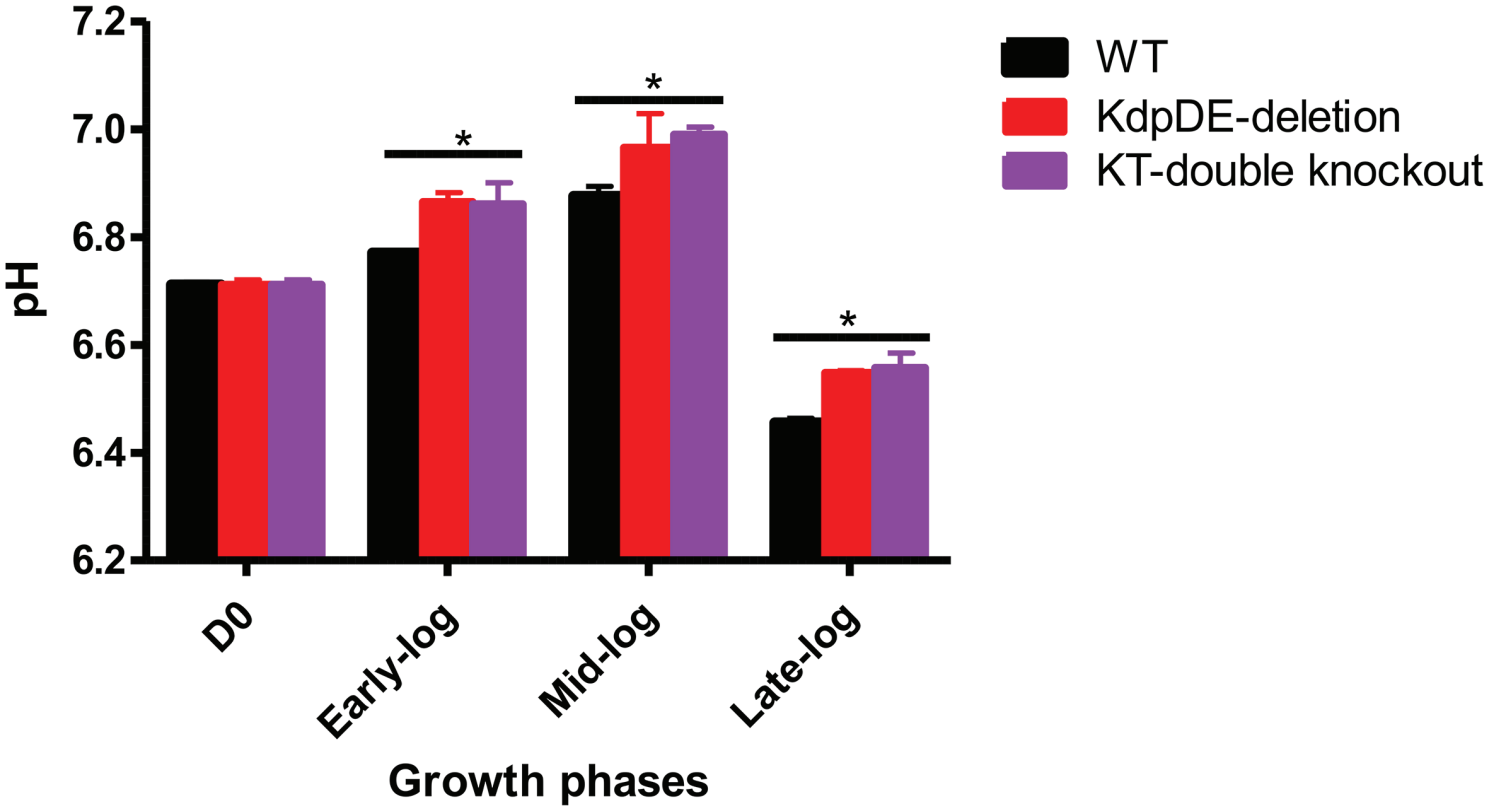
The extracellular pH levels at the three growth phases. The results are of three experiments performed in triplicate. The pH levels were similar at D0 for all the strains 6.711 ± 0.01. The pH levels for the WT, KdpDE-deletion and KT-double knockout mutant strains at early-log were: 6.77 ± 0.011, 6.865 ± 0.017, and 6.86 ± 0.04; at mid-log: 6.876 ± 0.045, 6.965 ± 0.064, and 6.99 ± 0.04; and at late-log: 6.455 ± 0.022, 6.548 ± 0.004, and 6.557 ± 0.028, respectively. Statistically significant differences between D0 vs. the log phases are shown with an (*) representing *p* < 0.05.

As shown in Figure 4, the extracellular pH values of the growth media increased at the early- to mid-log phases and decreased dramatically during the late-log phase, achieving statistical significance in comparison with the corresponding D0 pH value (pH 6.711) for all three strains. The extracellular pH levels of the two mutant strains were comparable at the three log phases of growth, but were higher than those of the WT strain.

### Gene Expression

The effects of the KdpDE regulatory system alone and in the presence of the Trk system on the expression of the K^+^-uptake genes during bacterial growth were explored by determining the expression levels of all six *kdp* and two *trk* genes in the WT and both mutant strains at the early-, mid-, and late-log growth phases in standard 7H9 liquid culture medium using RT-PCR. We confirmed mutations of the *kdpDE* (*kdpD* and *kdpE*) and *ceoBC* (*ceoB* and *ceoC*) genes by formation of non-specific fragments, with different melting temperatures from those of targeted gene-specific fragments using melting curve analysis data (Supplementary Table S2).

**Figure 5:**
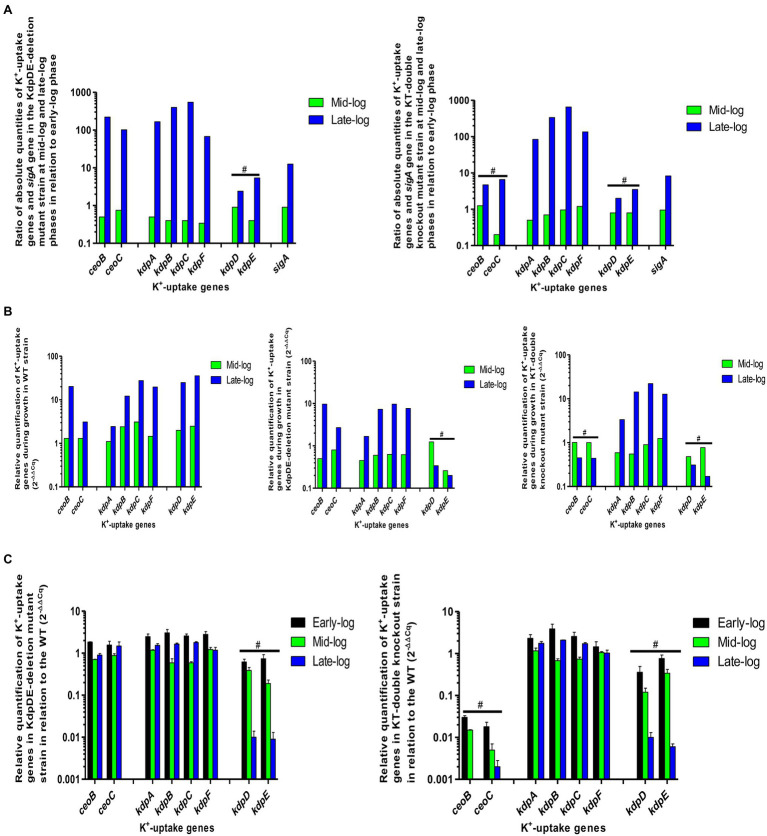
Gene expression measured during the various phases of bacterial growth for the WT and mutant strains. **(A)** Ratio of gene expression of absolute quantities of the K^+^-uptake genes and the *sigA* gene in the mutant strains during growth at mid- and late-log phases in relation to early-log phase, **(B)** relative quantification of K^+^-uptake genes in the mutant strains during growth relative to early-log phase (2^−ΔΔCq^), and **(C)** relative quantification of K^+^-uptake genes in mutant strains relative to the WT (2^−ΔΔCq^). ^#^represents mutations of the individual genes.

The results were analyzed as absolute amounts (μg/ml; AQ) and relative quantifications (RQ) using *sigA* as the reference gene (Figure 5; Supplementary Table S3). As shown in our previous study under similar conditions (Cholo et al., 2015), the expression levels of the *sigA* gene were comparable between the WT and the KdpDE-deletion and the KT-knockout mutants between the early- and mid-log phases. However, at the late-log phase, expression levels of *sigA* were significantly increased in all the strains, possibly indicative of low pH-induced stress. Similarly, all the K^+^-uptake genes in all the strains were elevated during the late-log phase of growth, highlighting responses to alterations in the environmental conditions in the growth medium. Despite this, the levels of increased expression of the *sigA* gene in the WT were much lower than those of the K^+^-uptake genes illustrating the constant expression of the *sigA* gene at the logarithmic phase. However, the levels of *sigA* were excessively high in the mutants, increasing by 2–2.5-fold relative to those of the WT at the late-log phase due to low pH stress (Cholo et al., 2015), which are clearly shown by AQ data (Figure 5A; Supplementary Tables S3.1–S3.3).

For each strain, the expression of all the measured K^+^-uptake genes during growth was determined by comparing the expression levels of each gene at the mid- and late-log phases relative to those at the early-log phase.

#### WT Strain

##### Absolute Quantification

The AQ data for the WT strain were the same as we have previously reported (Cholo et al., 2015). In summary, during growth, at the early- and mid-log phases, the *kdp* and the *trk* genes were expressed at minimum levels and were upregulated at the late-log phase, with the *ceoB* gene being the most prominently induced gene among all the K^+^-uptake genes, followed by *kdpD* and *kdpF* (Figure 5A; Supplementary Table S3.3). Despite upregulation of the K^+^-uptake genes, bacterial growth was slow at the late-log phase, due presumably to low extracellular pH levels (Cholo et al., 2015). Similar findings have been reported in *E. coli*, revealing that expression levels of the *trk* and the *kdp* genes are dependent on the extracellular pH (Epstein, 2003).

##### Relative Quantification

The RQ values of the genes in the WT strain were determined during growth at the mid- and late-log phases in relation to the early-log phase (Figure 5B; Supplementary Table S3.4). Expression of the *kdpDE* and *kdpBC* genes was significantly increased at the mid-log phase, while all the *kdp* genes were upregulated at the late-log phase. In the case of the *trk* genes, expression levels of both genes were unchanged at the mid-log phase, while both genes were upregulated at the late-log phase, particularly the *ceoB* gene.

#### KdpDE-Deletion Mutant Strain

##### Absolute Quantification

During growth, expression levels of all the *kdpFABC* and *trk* genes were minimal at the early- and mid-log phases. However, the *kdpFABC* genes were upregulated at early-log phase probably as a response to the decrease in extracellular pH (6.7), while they were downregulated at mid-log phase at elevated pH level of 6.9. These genes were significantly increased by ≥1,000-fold at the late-log phase at the lower pH of 6.5 (Figure 3; Supplementary Tables S3.1 and S3.3). In relation to the WT (Cholo et al., 2015), the *kdp* genes were increased, while the *trk* genes were decreased at early-log phase. Both *kdpFABC* and *trk* genes were downregulated at mid-log phase. However, with the exception of the *ceoB* gene, and similar to those of the WT, the expression levels of the *ceoC* and *kdpFABC* genes were significantly increased by at least 1.5 and up to 7-fold relative to those of the WT at the late-log phase.

##### Relative Quantification

During growth, all the *kdpFABC* and *trk* genes were downregulated at the mid-log phase and upregulated at the late-log phase (Figure 5B; Supplementary Table S3.4). In relation to the WT strain (Figure 5C; Supplementary Table S3.5), all the *kdpFABC* and *trk* genes were increased at the early-log phase, decreased at mid-log phase, and upregulated at the late-log phase, showing dependency on extracellular pH levels for their expression.

The AQ and RQ results appear to show that inactivation of the KdpDE system leads to dysregulation of both the *kdpFABC* and *trk* genes, resulting in constitutive expression of both operons.

#### KT-Double Knockout Mutant

Only the *kdpFABC* genes were evaluated as the *kdpDE* and *trk* genes were deleted in this mutant strain as shown by Tm of non-specific fragments due to mutations of the targeted genes (Supplementary Table S1).

##### Absolute Quantification

The expression levels and patterns of the *kdpFABC* genes were similar to those of the KdpDE-deletion mutant at the three growth phases (Figure 5A; Supplementary Tables S3.2 and S3.3).

##### Relative Quantification

During growth, the *kdpA* and *kdpB* genes were downregulated, while expression of the *kdpC* and *kdpF* genes remained unchanged at the mid-log phase (Figure 5B; Supplementary Table S3.4). All the *kdpFABC* genes were upregulated at the late-log phase. In relation to the WT strain (Figure 5C; Supplementary Table S3.6) and similar to the KdpDE-deletion mutant, all the *kdpFABC* genes were increased at the early-log phase, decreased at the mid-log phase, and upregulated at the late-log phase.

## Discussion

*Mycobacterium tuberculosis* is able to grow and adapt to varying environmental conditions. We have previously demonstrated that this bacterial pathogen grows exponentially at the mid-log phase at optimum K^+^ concentrations and pH levels (14–15 mM K^+^, pH 6.8–7.0), but is attenuated for growth, acquiring slow growth-to-dormant status at low pH levels (pH 5.5–6.0), despite maintaining elevated K^+^ concentrations at the late-log phase (Cholo et al., 2015).

Most bacteria adapt to varying extracellular K^+^ concentrations and pH levels by utilizing different K^+^-uptake transporters. Bacterial species, such as *E. coli* and *Salmonella* species, in which these K^+^ transporters have been extensively studied, utilize the Trk at elevated K^+^ concentrations and neutral pH levels and the Kup at low pH levels (pH 5.5), while they use the Kdp in K^+^-limiting conditions at neutral pH (Epstein, 2003; Liu et al., 2013). We have previously shown that *M. tuberculosis*, which encodes the Trk and Kdp as the predominant K^+^-uptake transporters (Cole et al., 1998), utilize the Trk system at mid-log (15 mM K^+^, pH 6.8; Cholo et al., 2006) and late-log phases (15 mM K^+^, pH 6.5; Cholo et al., 2015). However, in the case of the Kdp system, we have observed that the KdpFABC K^+^-uptake transporter is suppressed at mid-log while, similar to the Trk transporter, it is upregulated at the late-log phase (Cholo et al., 2015).

However, information on the differential utilization of these K^+^-uptake systems during bacterial growth is limited. This was investigated in the current study, in which we determined the role of the TCS KdpDE system in regulating the activities of the two K^+^-uptake transporters, the KdpFABC and Trk, during bacterial growth. We achieved this by first constructing the KT-double knockout mutant by transforming the *kdpDE*-deletion fragment constructed previously (Parish et al., 2003; Table 1) into the Trk-deletion mutant, also constructed previously (Cholo et al., 2006). Acquisition of the KdpDE-deletion and KT-double knockout mutant strains of *M. tuberculosis* were essential prerequisites to enable us to probe the involvement of the KdpDE system in harmonizing the activities of the KdpFABC and Trk transporters.

The findings of the current study revealed that the TCS KdpDE of *M. tuberculosis* is mechanistically involved in accelerating the rate of bacterial growth by shortening the duration of the early-log phase. This contention is supported by the observation that selective inactivation of the KdpDE system of *M. tuberculosis* caused attenuation of growth that was associated with prolongation of the early-log phase and delayed progression to the mid- and late-log phases, even in the presence of favorable extracellular K^+^ concentrations and near-neutral pH, both of which are conducive to exponential growth. Involvement of the KdpDE regulatory system during the exponential phase of bacterial growth was also evident, as demonstrated by the upregulation of both the *kdpD* and *kdpE* genes in the WT strain at the mid-log phase (Figure 5B; Supplementary Table S3.4). Other studies in *M. tuberculosis* and *M. smegmatis* have emphasized the essentiality of the *kdpE* gene during bacterial growth in standard growth conditions *in vitro* (Sassetti et al., 2003; Griffin et al., 2011; Ali et al., 2017).

As shown previously, *M. tuberculosis* cultured in these conditions utilizes the Trk system as the main K^+^-uptake transporter for growth (Cholo et al., 2006). Interestingly, however, in the current study, the growth of *M. tuberculosis* expressing an intact Trk system in the absence of KdpDE system (selective *kdpDE*-gene knockout mutant strain) was significantly attenuated, seemingly, implicating the KdpDE regulatory system in modulating the activity of the Trk system during growth. Not surprisingly, dual inactivation of the KdpDE and the Trk systems resulted in the most severe attenuation of growth relative to both the WT and KdpDE-deletion single knockout mutant. These observations not only underscore the interaction between these systems in promoting optimum bacterial growth, but also the seemingly key involvement of KdpDE in regulating the Trk, as well as the KdpFABC systems.

Using the ^86^Rb^+^ uptake procedure to determine bacterial K^+^-uptake efficiency, we demonstrated that during growth in optimal conditions, *M. tuberculosis* uses the KdpDE system to regulate K^+^ uptake by the KdpFABC and Trk transporters. In our previous studies, using the Trk-deletion mutant strain (Cholo et al., 2006, 2015), we demonstrated that at elevated K^+^ concentrations, bacteria utilize the low-affinity Trk system for K^+^ uptake, while the high-affinity Kdp system is suppressed, being induced as a back-up in the absence of Trk, conferring high K^+^-uptake efficiency on the Trk-deletion mutant (Cholo et al., 2006, 2015). The increase in K^+^-uptake efficiency of the KdpDE-deletion mutant strain in the presence of both KdpFABC and Trk K^+^-uptake transporters observed in the current study appears to demonstrate failure of the bacteria to differentially regulate the utilization of the two transporters, resulting in both systems being simultaneously operative. Excessive uptake of K^+^ is likely to result in dysregulation of bacterial cytoplasmic pH creating an intracellular environment unfavorable for cellular metabolism for growth.

However, at late-log phase (15 mM K^+^, pH 6.5), despite bacterial requirements of both the Kdp and Trk systems, the Trk has been shown to be the main K^+^-uptake transporter responsible for uptake of the cation. This contention is supported by the ^86^Rb^+^ uptake data, showing low K^+^-uptake efficiency of the WT in relation to the Trk-deletion mutant, together with gene expression data (AQ) in the WT strain showing that the *ceoB* gene is the most highly induced gene among all the K^+^-uptake genes at late-log phase (Cholo et al., 2015). In the current study, we have shown that in these conditions, as with optimal growth, the bacteria use the KdpDE regulatory system to regulate the activities and expression of both K^+^-uptake transporters. This has been demonstrated by the findings of increased uptake of ^86^Rb^+^ consistent with dysregulated, simultaneous, excessive functioning of both the Kdp and Trk K^+^-uptake transporters, in the absence of the KdpDE regulatory system.

These findings illustrate the constitutive activation of the KdpFABC transporter in the absence of its inducer KdpDE (Epstein, 2003; Steyn et al., 2003; Freeman et al., 2013; Agrawal and Saini, 2014). Similar findings have been demonstrated in previous studies in KdpDE mutant strains of *E. coli* (Asha and Gowrishankar, 1993; Sardesai and Gowrishankar, 2001; Epstein, 2003). These suggest a spontaneous induction of this operon in the absence of its inducer, or alternatively, the presence of an additional mechanism(s) of induction. While these have not been identified in *M. tuberculosis*, such mechanisms that bypass KdpDE have been described in *E. coli* and involve utilization of the histone-like nucleoid-structuring (H-NS) protein, thioredoxin 1, and thioredoxin reductase (Cole et al., 1998; Sardesai and Gowrishankar, 2001; Epstein, 2016). Although present in *M. tuberculosis*, the involvement of these mechanisms in activation of KdpFABC has not been described (Cole et al., 1998).

We do concede that our findings on the expression of these two mycobacterial K^+^-uptake transporters at various stages of growth in the absence of the TCS KdpDE system, are based on quantitation of their mRNA expression, which represents a potential limitation of our study. Nevertheless, we do believe that our findings demonstrate a novel and potentially important dual regulatory role of the KdpDE system in harmonizing the activities of the Trk and Kdp K^+^ transporters to ensure stringent control of cytoplasmic pH and growth. We also believe that these findings represent a platform that enables progression to additional confirmatory studies that would include target gene promoter expression using the β-galactosidase assay in this difficult and slow-growing pathogen.

**Figure 6:**
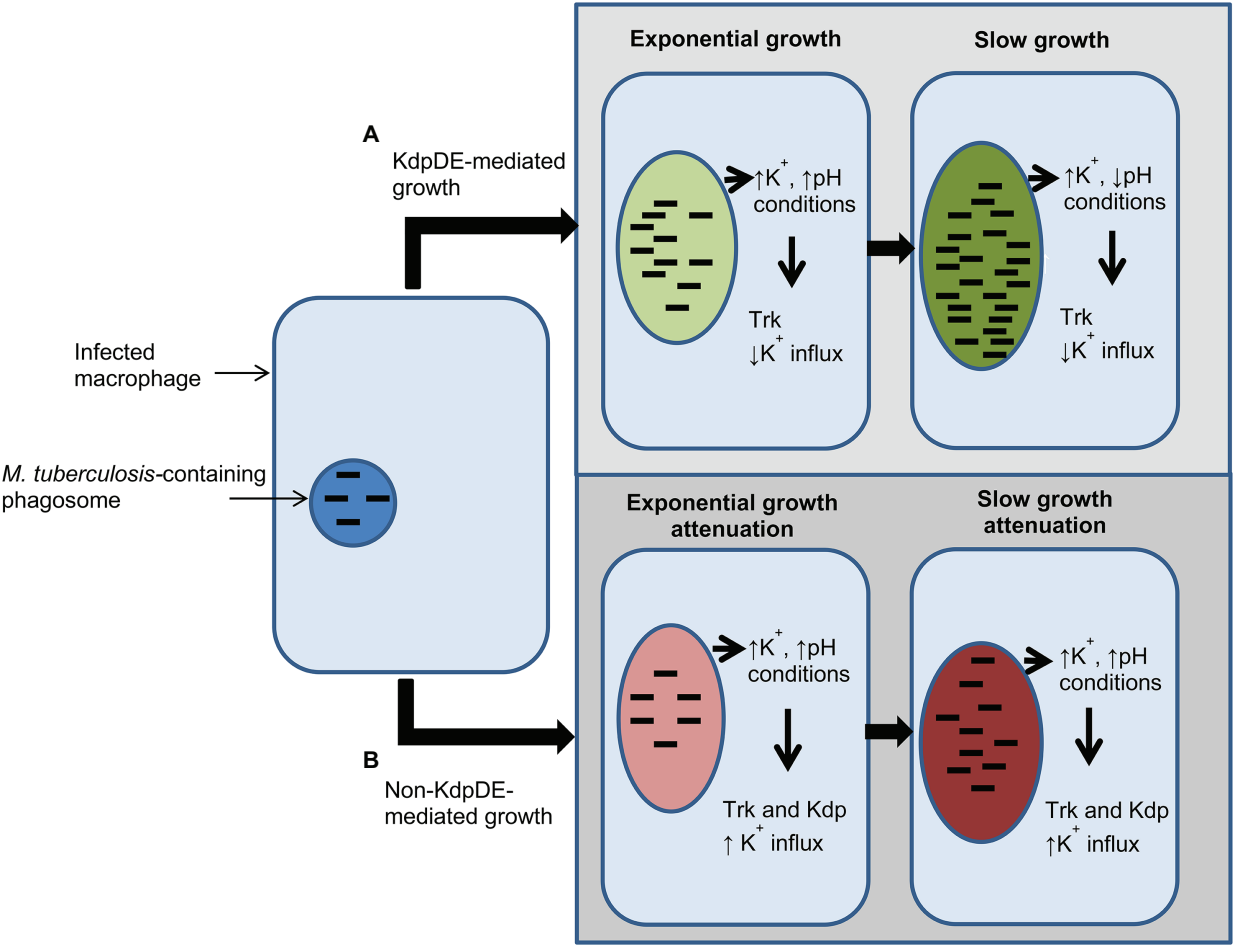
Schematic illustration summarizing events involved in bacterial growth in macrophages in the presence and absence of the two-component system (TCS) KdpDE. **(A)** In the presence of the KdpDE system, bacteria utilize the Trk transporter for K^+^ uptake suppressing the KdpFABC transporter, while **(B)** in the absence of the KdpDE both the KdpFABC and the Trk systems are constitutively activated, resulting in excessive influx of K^+^, which is detrimental to the bacteria, leading to attenuation of bacterial growth. ↑K^+^, phagosomal potassium concentration (19–50 mM); ↑pH, pH 6.7–6.8; ↓pH, pH 6.5.

These findings of the current study highlight the critical roles played by the K^+^-uptake transporters of *M. tuberculosis* during infection of the host. For example, in macrophages, the primary targets of the pathogen, in high intracellular K^+^ concentrations (phagosomal vacuolar K^+^ concentration: 19–50 mM; Wagner et al., 2005) and pH levels (pH 6.8; Piddington et al., 2000; Vandal et al., 2009), are probably conducive to bacterial growth. Presumably in this setting, the KdpDE and Trk systems are utilized by *M. tuberculosis* to establish infection (Haydel and Clark-Curtiss, 2004; Rengarajan et al., 2005; MacGilvary et al., 2019), possibly playing a role in bacterial virulence. However, at low pH levels, *M. tuberculosis* bacteria may utilize both the Kdp and Trk systems for survival (Figure 6). In this context, absence of the KdpDE system alone, and particularly in combination with the Trk system, is clearly detrimental to bacterial growth, underscoring the potential of these K^+^ transporters to serve as potential targets for development of anti-TB drugs.

In conclusion, in *M. tuberculosis*, the KdpDE system plays a key modulatory role in controlling the activities of the KdpFABC and Trk K^+^ uptake transporters to regulate growth.

## Data Availability Statement

The original contributions presented in the study are included in the article/Supplementary Material, further inquiries can be directed to the corresponding author.

## Author Contributions

MC and RA contributed to the conception and design of the study and wrote, edited and reviewed the manuscript. MC constructed the mutant strain. MC, MM, and AO performed the phenotypic experiments. MC, MM, AO, and RA contributed to interpretation of the data. All authors contributed to the article and approved the submitted version.

## Conflict of Interest

The authors declare that the research was conducted in the absence of any commercial or financial relationships that could be construed as a potential conflict of interest.

## Publisher’s Note

All claims expressed in this article are solely those of the authors and do not necessarily represent those of their affiliated organizations, or those of the publisher, the editors and the reviewers. Any product that may be evaluated in this article, or claim that may be made by its manufacturer, is not guaranteed or endorsed by the publisher.
