# Supplementary material for: Role of the kdpDE Regulatory Operon of Mycobacterium tuberculosis in Modulating Bacterial Growth in vitro
Source: Front Genet. 2021 Jul 29;12:698875. doi: 10.3389/fgene.2021.698875 (PMC8358298; doi:10.3389/fgene.2021.698875)
Supplement: Supplementary file 1 [file Data_Sheet_1.pdf]

Supplementary data

Table S1. Measurements of extracellular K^+ concentrations (mM) at different logarithmic growth phases

**Strain**	**7H9 broth unprocessed**	**7H9 broth processed**	**WT**	**KdpDE-deletion**	**KT-double knockout**
	14.15 ± 0.99	14.21 ± 1.01			
**D0**	NA		14.2 ± 1.024	14.21± 0.63	14.13 ±1.02
**Early-log**	NA		15.3 ± 0.08	15.2 ± 0.14	15.4 ± 0.09
**Mid-log**	NA		15.5 ± 0.7	15.18 ± 0.6	15.08 ± 0.6
**Late-log**	NA		14.6 ± 1.09	14.6 ± 1.02	14.8 ± 1.14

NA, Not applicable

Table S2. Melting curve analysis of the non-specific and targeted fragments

Gene name	Primer sets of all genes[a]		Target gene-specific fragment length (bp)[a]	Melting temperature (Tm)	
	Forward primer	Reverse primer		Mutation/ non-specific, no template control ± SD	Target gene-specific fragment ± SD
ceoB	CGG CGA CAA CTC CAA CAT (18)	CGG CAC ACC GAA GGT TTC (18)	58	79.9 ± 0.31	86.2 ± 0.25
ceoC	CTG CTG GAG TCG ATT CAC CT (20)	ACC GCG AAT TCG GTC TTG (20)	107	83.1 ± 0.22	91.2 ± 0.23
kdpA	GAT TGA ACG GCC TAC TGG TC (20)	CTG GAT CTT CTT GCC GAG AT (20)	95	80.2 ± 0.35	87.5 ± 0.22
kdpB	CTG GGC TGA CGA TCA TCT TT (20)	CGT GGT CGG AAT GAG ACA C (19)	122	79.4 ± 0.11	90.6 ± 0.21
kdpC	TAC CGC AAG GAA AAC AAT CTG (21)	GGC ATT GAC CAC CGA TATG (19)	102	79.2 ± 0.28	90.2 ± 0.25
kdpF	AAC ATC GTC GGG TTG GTG (18)	GCG AAT AGG AAC GCC ATT AG (20)	50	79.3 ± 0.3	83.5 ± 0.23
kdpD	AGT CCA TCG ACC AAC TCA CC (20)	CAC CGC TTC CTC CAG GTA T (19)	110	78.4 ± 0.15	91.4 ± 0.22
kdpE	TGG AAT GGA CGA GTT TCT GG (20)	GAC GGT GAA TGA ATC GGT TT (20)	103	79.9 ± 0.31	91.5 ± 0.23
sigA	CAA GGA CGC CGA ACT CAC (18)	CTT GCC GAT CTG TTT GAG GT (20)	64	78.9 ± 0.24	87.4 ± 0.23

a, Information is as described (Cholo et al., 2015).

Table S3. Gene expression measured during the various phases of bacterial growth for the WT and mutant strains.

Absolute quantification

Table S3.1. Absolute quantification of K^+-uptake gene in KdpDE-deletion knockout mutant

Gene name	Early-log ± SD (μg/mL)	Mid-log ± SD (μg/mL)	Late-log ± SD (μg/mL)
ceoB	15.37 ± 4.3	7.15 ± 0.7	3325 ± 1068
ceoC	19.7 ± 1.96	15.32 ± 0.66	2032 ± 623.23
kdpA	26.37 ± 4.3	11.78 ± 2.49	4346.1 ± 1648.1
kdpB	8.59 ± 3.19	4.08 ± 1.48	3597.43 ± 1421.3
kdpC	4.56 ± 1.54	2.17 ± 0.19	2738.96 ± 796.2
kdpF	34.8 ± 9.8	11.88± 3.64	2375.25 ± 646.71
kdpD	*30.7 ± 2.49*#	*28.75 ± 3.1*#	*73.44 ± 14.69*#
kdpE	*5.14 ± 0.65*#	*2.38 ± 0.62*#	*27.1 ± 10.57*#
sigA	204.3 ± 17.94	181.88 ± 7.44	2545 ± 801.43

Italised numbers represent mutations of the individual genes

Table S3.2. Absolute quantification of K^+-uptake gene in KT-double knockout

**Gene name**	**Early-log ± SD (µg/mL)**	**Mid-log ± SD (µg/mL)**	**Late-log ± SD (µg/mL)**
ceoB	*0.43 ± 0.06*#	*0.485 ± 0.05*#	*2.04 ± 0.23*#
ceoC	*0.39 ± 0.3*#	*0.07 ± 0.06*#	*2.59 ± 0.64*#
kdpA	26.17 ± 2.98	13.69 ± 1.153	2190.63 ± 570.36
kdpB	8.91 ± 2.36	5.57 ± 1.47	3036.75 ± 670.87
kdpC	3.51 ± 0.47	3.38 ± 0.28	2297.25 ± 507.8
kdpF	13.91 ± 3.1	17.08 ± 7.12	1891.88 ± 378.04
kdpD	*31.68 ± 3.014*#	*27.3 ± 3.38*#	*63.6 ± 11.88*#
kdpE	*5.81 ± 0.54*#	*4.46 ± 0.27*#	*21.25 ± 5.23*#
sigA	215 ± 12.24	204.88 ± 8.501	1774.63 ± 342.5

Italised numbers represent mutations of the individual genes

Table S3.3. Ratio of gene expression of absolute quantities of the K^+-uptake genes and the *sigA* gene in the mutant strains during growth at mid- and late-log phases in relation to early-log phase

Gene name	KdpDE-deletion mutant		KT-double knockout mutant	
	Mid-log phase	Late-log phase	Mid-log phase	Late-log phase
ceoB	0.5	222	*1.25*#	*4.7*#
ceoC	0.75	102	*0.2*#	*6.6*#
kdpA	0.5	167	0.5	84
kdpB	0.4	400	0.7	337
kdpC	0.4	548	0.96	656
kdpF	0.34	68	1.2	135
kdpD	*0.9*#	*2.4*#	*0.8*#	*2*#
kdpE	*0.4*#	*5.4*#	*0.8*#	*3.5*#
sigA	0.9	12.5	0.95	8.3

#italised numbers represent mutations of the individual genes

Relative quantification

Table S3.4. Relative quantification of K^+-uptake genes in the mutant strains during growth ($2^{-\Delta\Delta Cq}$)

Gene name	WT		KdpDE-deletion mutant		KT-double knockout mutant	
	Mid-log phase	Late-log phase	Mid-log phase	Late-log phase	Mid-log phase	Late-log phase
ceoB	1.3	20.3	0.5	9.71	*1.01*#	*0.45*#
ceoC	1.3	3.1	0.8	2.7	*909*#	*0.44*#
kdpA	1.1	2.44	0.45	1.67	0.59	3.36
kdpB	2.4	12.2	0.6	7.3	0.55	14.3
kdpC	3.1	27.7	0.63	9.7	0.9	22.2
kdpF	1.46	19.7	0.62	7.67	1.25	12.8
KdpD	1.99	24.9	*1.24*#	*0.34*#	*0.48*#	*0.31*#
KdpE	2.48	35.5	*0.26*#	*0.2*#	*0.77*#	*0.17*#

Italised numbers represent mutations of the individual genes

Table S3.5. Relative quantification of K^+-uptake genes of KdpDE-deletion mutant relative to the WT with confidence interval (CI)

Gene name	Early-log ± SD ($2^{-\Delta\Delta Cq}$)	CI	Mid-log ± SD ($2^{-\Delta\Delta Cq}$)	CI	Late-log ± SD ($2^{-\Delta\Delta Cq}$)	CI
ceoB	1.84 ± 0.03	1.8-1.9	0.71 ± 0.01	0.68-0.73	0.9 ± 0.08	0.71-1.1
ceoC	1.57 ± 0.36	0.66-2.49	0.88 ± 0.09	0.65-1.11	1.48 ± 0.38	0.5-2.46
kdpA	2.49 ± 0.38	1.52-3.46	1.18 ± 0.04	1.1-1.28	1.54 ± 0.15	1.15-1.92
kdpB	3.06 ± 0.61	1.47-4.61	0.59 ± 0.15	0.21-0.97	1.65 ± 0.1	1.41-1.89
kdpC	2.58 ± 0.29	1.82-3.33	0.59 ± 0.04	0.49-0.68	1.8 ± 0.08	1.54-2.06
kdpF	2.8 ± 0.47	1.59-4.01	1.23 ± 0.14	0.87-1.6	1.18 ± 0.2	0.66-1.69
kdpD	*0.62 ± 0.1*#	*0.35-0.89*	*0.39 ± 0.07*#	*0.21-0.57*	*0.01 ± 0.004*#	*0.001-0.02*
KdpE	*0.74 ± 0.18*#	*0.29-1.2*	*0.19 ± 0.04*#	*0.08-0.31*	*0.009 ± 0.004*#	*-0.0004-0.018*

Italised numbers represent mutations of the individual genes

Table S3.6. Relative quantification of the K-uptake genes of KT-double knockout mutant relative to WT with confidence intervals (CI)

Gene name	Early-log ratio ± SD	CI	Mid-log ± SD	CI	Late-log ± SD	CI
ceoB	*0.03 ± 0.003*#	*0.01-0.03*	*0.015 ± 0.0003*#	*0.014-0.016*	*0.0006 ± 0.0002*#	*0.00005-0.0011*
ceoC	*0.018 ± 0.005*#	*0.005-0.032*	*0.005 ± 0.002*#	*-0.0009-0.01*	*0.002 ± 0.0008*#	*0.0002-0.004*
kdpA	2.32 ± 0.52	0.97-3.66	1.16 ± 0.19	0.68-1.63	1.76 ± 0.18	1.17-2.34
kdpB	3.87 ± 1.17	0.85-6.89	0.68 ± 0.07	0.49-0.86	2.1 ± 0.02	2.06-2.2
kdpC	2.56 ± 0.66	0.86-4.26	0.74 ± 0.08	0.52-0.96	1.72 ± 0.11	1.43-2.00
kdpF	1.45 ± 0.46	0.25-2.64	1.06 ± 0.06	0.90-1.22	1.03 ± 0.18	0.56-1.5
kdpD	*0.36 ± 0.13*#	*0.004-0.71*	*0.12 ± 0.03*#	*0.04-0.2*	*0.01 ± 0.003*#	*0.0018-0.018*
kdpE	*0.76 ± 0.16*#	*0.34-1.18*	*0.34 ± 0.08*#	*0.1-0.59*	*0.006 ± 0.001*#	*0.002-0.01*

Italised numbers represent mutations of the individual genes
